# Evaluation of digital thermography imaging to assess and monitor treatment of police working dogs with naturally occurring hip osteoarthritis

**DOI:** 10.1186/s12917-021-02876-z

**Published:** 2021-05-01

**Authors:** J. C. Alves, A. Santos, P. Jorge, C. Lavrador, L. Miguel Carreira

**Affiliations:** 1 Divisão de Medicina Veterinária, Guarda Nacional Republicana (GNR), Rua Presidente Arriaga, 9, 1200-771 Lisbon, Portugal; 2 MED – Mediterranean Institute for Agriculture, Environment and Development, Instituto de Investigação e Formação Avançada, Universidade de Évora, Pólo da Mitra, Ap. 94, 7006-554 Évora, Portugal; 3 Faculty of Veterinary Medicine, University of Lisbon (FMV/ULisboa), Lisbon, Portugal; 4 Interdisciplinary Centre for Research in Animal Health (CIISA) – University of Lisbon, (FMV/ULisboa), Lisbon, Portugal; 5 Anjos of Assis Veterinary Medicine Centre (CMVAA), Barreiro, Portugal

**Keywords:** Dog, Osteoarthritis, Hip, Digital thermography, Stance analysis, Clinical metrology instruments

## Abstract

**Background:**

In dogs, thermal imaging has been documented only recently, but a growing interest in this modality has led to studies using thermography to assess pathologies in the canine hip, stifle, elbow, intervertebral disc, and bone neoplasia. This study aimed to evaluate the use of digital thermography in assessing and evaluating treatment response in dogs with hip osteoarthritis (OA) and comparing its results with an objective measure and two clinical metrology instruments. In an experimental, randomized, double-blinded study, one hundred hip joints of fifty police working dogs with bilateral hip OA were evaluated. A dorsoventral and lateral thermographic image were obtained on days 0, 8, 15, 30, 90, and 180. Mean and maximal temperatures were determined. Additionally, the animal’s weight-bearing distribution and radiographic examination of the hip joint (extended legs ventrodorsal view) were performed. Copies of the Canine Brief Pain Inventory (CBPI) and Canine Orthopaedic Index (COI) were obtained. Results were analyzed by ANOVA, followed by an LSD post-hoc test, and correlations were assessed with Spearman correlation coefficient, with *p* < 0.05.

**Results:**

Values recorded on the lateral view were higher than those on the dorsoventral view. No differences or correlations were found between Orthopedic Foundation for Animals hip grades and temperature. Digital thermographic images showed a weak significant correlation with weight-bearing evaluations (*r* = 0.13, *p* < 0.01) and different clinical metrology instruments scores (*r* = − 0.25, *p* < 0.01 for pain severity score, and *r* = − 0.21, *p* = 0.04 for gait). It also correlated with radiographic findings, specifically the circumferential femoral head osteophyte and caudolateral curvilinear osteophyte.

**Conclusion:**

To our knowledge, this is the first study presenting the digital thermography assessment of Police working dogs submitted to treatment for hip OA. Digital thermography, mainly based on a lateral view evaluation, showed a weak significant correlation with stance analysis and clinical metrology instruments scores.

## Background

Digital thermography is a contact-free, non-invasive screening tool that can assess soft tissue injuries, including muscle strains, sprains, tendinopathies, and OA in humans, horses, cats, and dogs [1–3]. It relies on identifying changes in heat in tissue due to disruptions of tissue morphology and physiological functions, which in turn relate to skin temperature control [4–6]. The rationale behind its use is that an injury is often associated with variations in blood flow on the affected site, changing the skin temperature [7]. Changes in blood flow rate, local structures of subcutaneous tissues, and the sympathetic nervous system’s activity are reflected on skin temperature through a complex system [1]. Inflammation in subcutaneous and deeper tissues reflects temperature changes in superficial tissues. These changes are a product of the inflammation mechanism, which influences blood vessels’ diameter, blood flow rate, and capillary permeability [8, 9]. Digital thermography can provide a reproducible screening tool by describing the specific changes in each disease process [10, 11]. It has been described as useful in humans, horses, and cats [1, 3, 12, 13]. There has been a growing interest in thermal imaging in dogs, with recent reports on thermography use to assess pathologies in the canine hip, stifle, elbow, intervertebral disc, and bone neoplasia published [2, 13–18]. These studies have described normal thermal imaging of different body areas or diseases. Still, there are no studies available to our knowledge comparing the results of thermal imaging with other evaluation modalities and in the evaluation of response to treatment.

OA is the most prevalent joint disease in dogs, with an estimated prevalence of 20% [19–22]. Since the disease lacks obvious extra-articular manifestations, it is well suited to use a local therapy by intra-articular (IA) injection [23]. Commonly used IA treatment modalities include corticosteroids (as triamcinolone hexacetonide), hyaluronan, and autologous platelets [24–26]. Pain is the most relevant clinical sign of OA and a hallmark of the disease [27, 28]. Several clinical metrology instruments have been developed to assess pain and evaluate treatment response [29]. One of the best clinical metrology instrument created for dogs is the Canine Brief Pain Inventory (CBPI), divided into a pain severity score (PSS), to evaluate the overall pain magnitude and a pain interference score (PIS), to assesses the degree to which pain affects daily activities [30–34]. The Canine Orthopaedic Index (COI) is an additional clinical metrology instrument to evaluate other dimensions of OA’s impact. It is divided into stiffness, gait, function, and quality of life scores [35–38]. A typical assessment performed during the orthopedic examination is evaluating weight distribution, off-loading, or limb favoring at the stance [39]. OA patients exhibit subtle shifts in body weight distribution at a stance due to pain or instability [40, 41]. Stance analysis, which evaluates individual limb weight-bearing, is an objective measure, reported as sensitive for detecting lameness in dogs [41], and equivalent or superior measurement of hip OA-related pain associated with hip OA than vertical impulse and peak vertical force [41].

This study aimed to evaluate the use of digital thermography in assessing and evaluating treatment response in dogs with hip OA. As OA is a disease involving multiple dimensions, from changes in limb function, ability to conduct daily activities, and demeanor [32], we also aimed to compare the results of digital thermography to an objective measure (weight-bearing evaluation) and two clinical metrology instruments (the CBPI and the COI). We hypothesize that digital thermography results will correlate with the weight-bearing assessment results, the considered clinical metrology instruments, and radiographic examination.

## Results

The sample from this study included 100 limbs of 50 Police working dogs with bilateral hip OA: 17 German Shepherd Dogs, 15 Belgian Malinois Shepherd Dogs, 10 Labrador Retriever, and 8 Dutch Shepherd Dog, 30 from males and 20 females, with a mean age of 6.5 ± 2.2 years, bodyweight of 26.7 ± 5.3 kg and body condition score of 4/9 [42]. Joints were classified as mild (*n* = 70), moderate (20), and severe (10), according to the Orthopedic Foundation for Animals hip grading scheme [43, 44]. Three images (1 DV and 2 LT, to have a DV and LT view from each joint) were obtained from each animal in six different evaluation moments (days 0, 8, 15, 30, 90, and 180), amounting to 900 images. All patients were followed up to the last evaluation day. At the initial evaluation (day 0), the mean and maximal temperature on the DV were 24.7 °C ± 1.7 and 25.8 °C ± 1.7, respectively. On the LT view, mean and maximal temperatures were 26.1 °C ± 2.3 and 28.1 °C ± 2.4, respectively. The thermographic evaluation results in each view by the Orthopedic Foundation for Animals hip grades are presented in Table 1. No significant differences or correlations were found between OFA hip grade and temperature.

**Table 1:** Mean and maximal thermographic evaluation values (±standard deviation) of ventrodorsal and lateral views, by Orthopedic Foundation for Animals hip grades at the initial evaluation

OFA hip grade	Dorsoventral view		Lateral view	
	(°C, mean ± SD)		(°C, mean ± SD)	
	Mean	Max	Mean	Max
Mild (*n* = 70)	24.9 ± 1.6	25.9 ± 1.6	26.2 ± 2.2	28.2 ± 2.1
Moderate (*n* = 20)	24.9 ± 1.8	25.9 ± 1.9	26.1 ± 2.6	28.2 ± 2.4
Severe (*n* = 10)	24.0 ± 1.8	25.0 ± 1.7	25.5 ± 2.6	27.2 ± 2.3

Mean and maximal values of ventrodorsal and lateral views on each evaluation day are presented in Table 2. Compared to the initial evaluation, significant variations in the thermographic evaluation were recorded mainly on the lateral view. During follow-up evaluations, significant differences in the result of treatment were registered with SI (*p* < 0.01) and deviation (*p* < 0.01). Correlations between thermography evaluation and weight-bearing evaluation, on the initial assessment and during the follow-up period, are presented in Table 3. Correlations between thermography evaluation and clinical metrology instrument scores on the first assessment and during the follow-up period are shown in Table 4. Considering radiographic findings, at the initial evaluation, maximal DV, mean and maximal LT thermographic evaluations showed a weak correlation with the presence of caudolateral curvilinear osteophyte on the ventrodorsal view (*r* = − 0.2, *p* = 0.05; *r* = − 0.3, *p* < 0.01 and *r* = − 0.2, *p* = 0.04, respectively).

**Table 2:** Mean and maximal thermographic evaluation temperature values (±standard deviation) of ventrodorsal and lateral views, on each evaluation moment. * indicates significant differences when compared with day 0 evaluation (*p* < 0.01). Negative values represent negative correlations between values

Instant	Dorsoventral view		Lateral view	
	(°C, mean ± SD)		(°C, mean ± SD)	
	Mean	Max	Mean	Max
0	24.9 ± 1.7	25.8 ± 1.7	26.1 ± 2.3*	28.1 ± 2.4*
8	24.0 ± 2.3	25.5 ± 2.2	30.9 ± 2.3*	34.6 ± 1.6*
15	26.6 ± 2.2	25.9 ± 2.3	29.5 ± 3.1*	34.3 ± 1.6*
30	24.8 ± 2.3	26.3 ± 2.3	29.6 ± 2.6*	33.6 ± 1.9*
90	25.8 ± 1.3	27.1 ± 1.4*	28.5 ± 2.1*	30.7 ± 2.3*
180	25.5 ± 1.3	26.9 ± 1.2*	28.2 ± 2.1*	30.6 ± 2.1*

**Table 3:** Correlation coefficients between thermography evaluation and weight-bearing evaluation (symmetry index and deviation from the normal 20% weight-bearing), on the initial evaluation and during the follow-up period. COI – Canine Orthopedic Index; PIS – Pain Interference Score; PSS – Pain Severity Score; QOL – Quality of Life; SI – Symmetry Index. ^a^ indicates a significant correlation

		Initial evaluation		Follow up period	
Measure		SI	Deviation	SI	Deviation
Dorsoventral mean	r_s_	0.22	0.06	−0.05	−0.03
	Sig.	0.83	0.59	0.27	0.49
Dorsoventral max	r_s_	0.48	0.04	−0.12	−0.05
	Sig.	0.65	0.73	0.01^a^	0.27
Lateral mean	r_s_	0.04	0.05	0.08	0.09
	Sig.	0.97	0.65	0.08	0.04^a^
Lateral max	r_s_	0.03	0.07	0.13	0.10
	Sig.	0.75	0.51	< 0.01^a^	0.04^a^

**Table 4:** Correlation coefficients between thermography evaluation and clinical metrology instrument scores, on the initial evaluation and during the follow-up period. COI – Canine Orthopedic Index; PIS – Pain Interference Score; PSS – Pain Severity Score; QOL – Quality of Life; SI – Symmetry Index. ^a^ indicates a significant correlation

Evaluation moment	Measure		Score						
			PSS	PIS	COI	Stiffness	Function	Gait	QOL
**T0**	Dorsoventral mean	r_s_	−0.01	−0.03	−016	−0.21	−0.11	−0.22	−0.03
		Sig.	0.89	0.78	0.13	0.04^a^	0.28	0.04^a^	0.78
	Dorsoventral maximal	r_s_	−0,02	−0.03	−0.14	− 0.17	−0.11	− 0.19	−0.01
		Sig.	0.89	0.81	0.19	0.10	0.31	0.06	0.97
	Lateral mean	r_s_	−0.04	−0.06	−0.12	− 0.10	−0.41	− 0.21	−0.02
		Sig.	0.73	0.59	0.28	0.32	0.69	0.04^a^	0.84
	Lateral maximal	r_s_	−0.01	−0.01	−0.09	− 0.84	−0.07	− 0.17	0.03
		Sig.	0.99	0.98	0.36	0.42	0.48	0.09	0.81
**Follow up period**	Dorsoventral mean	r_s_	−0.04	−0.04	−0.06	− 0.05	−0.04	− 0.06	−0.07
		Sig.	0.42	0.36	0.25	0.32	0.38	0.23	0.16
	Dorsoventral maximal	r_s_	0.06	−0.01	−0.01	0.02	0.01	−0.06	−0.01
		Sig.	0.23	0.91	0.77	0.70	0.91	0.22	0.97
	Lateral mean	r_s_	−0.10	−0.01	0.04	0.03	0.06	0.01	0.08
		Sig.	0.03^a^	0.82	0.37	0.59	0.22	0.79	0.08
	Lateral maximal	r_s_	−0.25	−0.06	−0.01	−0.01	− 0.01	−0.1	0.3
		Sig.	< 0.01^a^	0.22	0.94	0.86	0.79	0.79	0.5

## Discussion

Digital thermal imaging can be used to assess musculoskeletal conditions, including OA, based on the variations in blood flow that injury and inflammation generate, which can affect the skin temperature [7–9]. To our knowledge, this is the first study to describe the use of digital thermography in the initial evaluation and to monitor treatment outcome in dogs with OA.

Pain is the most relevant clinical sign of OA [27, 28], and it is a multi-dimensional experience with functional, sensory, evaluative, and affective components [45]. To capture information regarding this wide-ranging nature of the disease, we choose to compare digital thermography to an objective measure, stance analysis [41], directed at evaluating the function, and two different clinical metrology instruments, to assess pain and the ability to conduct specific daily activities. Previous reports indicate that SI is reliable indicators of clinical lameness in dogs [46]. Considering a cut-off point of 18% of weight-bearing for pelvic limbs seems to increase sensitivity and specificity [41, 47, 48]. For that reason, we compared thermography scores with both SI and deviation from the normal value of 20%. During the follow-up period, digital thermography results based on the lateral view, specifically the maximal value, showed low but significant correlations with stance analysis results, which provides evidence favoring using a lateral view rather than a dorsoventral when monitoring OA treatment. Still, the dorsoventral maximal value also showed a week significant correlation with SI. This is not entirely unexpected, as inflammatory mediators drive OA within the joint, and thermography has shown to be a reliable technique to assess inflammatory pain and differentiate normal from human osteoarthritis patients [49–51]. The use of the maximal temperature value may better reflect this inflammatory process and nature. It may also present an additional advantage for less experienced operators than mean values. This last approach requires a more precise determination of anatomical areas of interest and is influenced by incorrect inclusion of measurements from non-affected tissues. It is also well established that the perception of pain and overall joint function is influenced by sensory innervation of the tissues that compose the joint, from the subchondral bone, periosteum, synovium to the capsule, and surrounding tissues, such as muscles [52, 53]. As LT views include a more significant amount of muscle masses than DV views, which are also involved in the disease process and under inflammation, this may account for higher mean and maximal temperature values registered on a lateral view [51, 54]. It may also account for the weak significant correlation between LT evaluations and pain severity scores during the follow-up evaluations. An additional reason for these weak but significant correlations (also reflected in the scatter plot) may be related to the fact that different evaluation methods are capturing different dimensions of OA [32].

The thermographic evaluation also correlated with clinical metrology instruments scores, specifically gait and function, at the initial assessment. This can be explained by the fact that inflammation, whose effect is recorded by digital thermography, reflects affected tissues, their contribution to pain perception, and loss of normal function. These signs, related to an inability to perform normal daily activities, are most likely to present a patient for consultation [29]. As most joints represented in this sample were classified as mild OA, they are likely expressed mainly on scores that aim to measure functionality rather than overall demeanor, such as QOL. A weak significant correlation was observed with the PSS, but not with PIS, and only during the follow-up period. This is not entirely unexpected, as patients in this study high-drive working dogs, which tend to be stoic and usually show only subtle signs of pain, making its evaluation more challenging [55, 56].

In a human OA study, increased temperatures have been related to even slight degenerative changes and low temperatures with more severe disease cases [9]. In other reports, a correlation between increasing temperature and more severe radiographic changes has been described [57, 58]. Although no differences were found in the thermographic evaluation of different OFA hip grades, hip joints classified as severe did have lower values in all considered thermographic evaluations, which may signal a trend. With severe OA, a loss of the tissues that surround the joint also occurs [1, 51]. These factors may be responsible for the decrease in temperature observed in severe hip grades than moderate hip grades. The evaluation of more hips classified as severe would help clarify this fact, as the large majority of joints considered in this study were classified as mild. The circumferential femoral head osteophyte and the caudolateral curvilinear osteophyte are the two features that represent early radiographic signs that predict the development of the clinical symptoms of hip OA [43, 59–61]. This is supported by our results, as both showed a correlation with digital thermography evaluation and may be linked to the inflammatory process that drives OA and is responsible for producing clinical signs.

This study evaluated digital thermography’s ability to assess and assess treatment response in dogs with hip OA by comparing digital thermography results with other commonly used and validated evaluation modalities. A limitation of the study is related to the fact that evaluated dogs were working dogs, which tend to be stoic, making it more challenging to assess these patients using the CBPI [55, 56]. Also, the majority of animals had mild or moderate OA. It would be of interest to include disease-free patients and a larger proportion of animals representing the remaining hip grades to describe their digital thermography evaluation and this evaluation technique’s ability to differentiate between them.

## Conclusions

To our knowledge, this is the first study presenting the digital thermography assessment of Police working dogs submitted to treatment for hip OA. Significant variations were observed in the thermographic evaluation of patients between initial and follow-up evaluations. Digital thermography, mainly based on an LT view evaluation, correlated with weight-bearing distribution and clinical metrology instruments scores. It also correlated with the presence of caudolateral curvilinear osteophyte on the ventrodorsal view at the initial assessment, a finding associated with the development of clinical symptoms of hip OA. Digital thermography may be an option for the screening of dogs with hip OA.

## Methods

The sample comprised one hundred (*N* = 100) hip joints from fifty active police working dogs with naturally occurring bilateral hip OA, constituting a convenience sample of patients presenting for treatment in the Clinica Veterinária de Cães of the Guarda Nacional Republicana (Portuguese Gendarmerie), after they were first diagnosed. All patients were active working dogs and remained in active work during and after this study’s conclusion. For this report, data from a longitudinal double-blinded, negative controlled study was used. Animals were signaled based on a diagnosis consistent with bilateral hip OA. For the diagnosis, the dog’s history was considered, in addition to a difficulty to perform specific exercises (as rising, jumping, or maintaining obedience positions, leading to a worse performance), a physical examination consistent with hip OA (joint pain and stiffness, with a reduced range of motion), and an OFA hip scores of mild, moderate or severe. Additionally, they should be over 2 years and have a bodyweight ≥20 kg [62, 63]. They could not be on any medication or nutritional supplements for 6 weeks or more to allow a washout period [64]. Animals with any other suspected or documented orthopedic, neurological, or concomitant disease were excluded. Other conditions were ruled out through physical and radiographic examination, complete blood count, and serum biochemistry. The same researcher examined all patients.

Using a statistical analysis software, patients were randomly assigned to a group and, on day 0 (treatment day), either received an intra-articular administration of 0.9% NaCl (control group) or treatment (a platelet concentrate - VPET®, Hylan G-F 20, stanozolol or triamcinolone hexacetonide), the same to both hips, according to the assigned group. All groups had the same number of joints (*n* = 20), and all administrations were performed by the same researcher, blinded to the given group. No other medications/treatments were administered during the follow-up period.

**Fig. 1:**
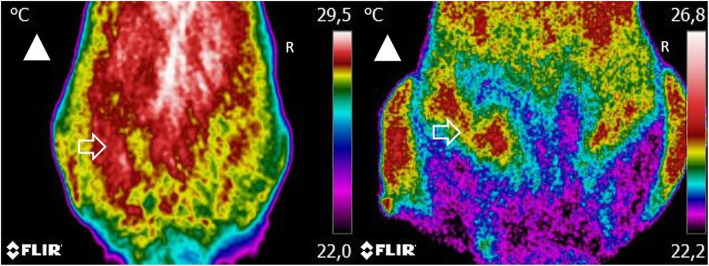
A dorsoventral view of two dogs with moderate (left) and severe (right) osteoarthritis. Images include the area from the last lumbar vertebra to the first coccygeal vertebra at a minimum, at a distance of 60 cm. Arrowhead indicates the direction of the dog’s head. Arrow indicates the anatomical location of the hip joint. Increased temperature areas are observed on the hip joint, but with surrounding lower temperatures on the patient with severe OA

**Fig. 2:**
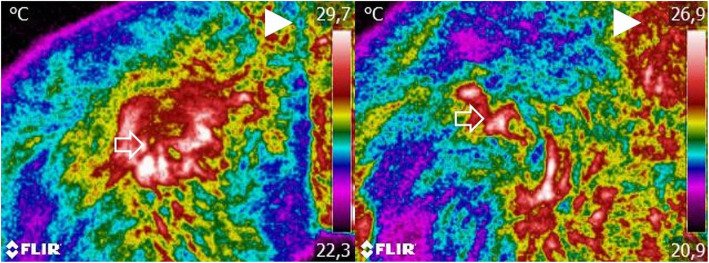
A dorsoventral view of the same two dogs represented in Fig. 1, with moderate (left) and severe (right) osteoarthritis. Images were taken with the greater trochanter in the center of the image at a distance of 60 cm. Arrowhead the direction of the dog’s head. Arrow indicates the anatomical location of the hip joint. Increased temperature areas are observed on the hip joint, but with surrounding lower temperatures on the patient with severe OA

**Fig. 3:**
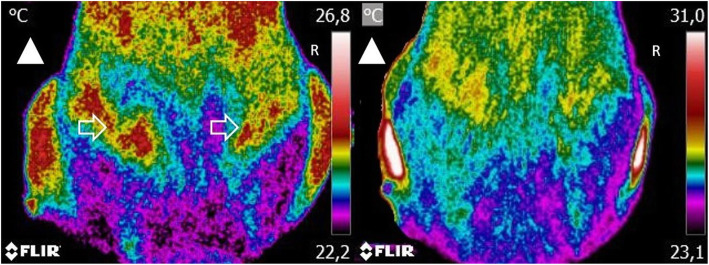
A dorsoventral view of the same dog at the time of initial evaluation (left) and 30-days post-treatment (right). Arrowhead the direction of the dog’s head. Arrow indicates the anatomical location of the hip joint. Arrow indicates the anatomical location of the hip joint. Lower temperatures are observed at 30-days post-treatment

Thermographic images, CBPI, COI, and weight-bearing evaluation results were recorded on days 0, 8, 15, 30, 90, and 180, corresponding to the day when the IA treatment response was evaluated. These evaluations were conducted before the IA administration. On each assessment, three images were taken sequentially: a dorsoventral view, a right lateral view, and a left lateral view, with a FLIR ThermaCAM E25® camera. A single researcher, blinded to the patient’s assigned group, performed the thermographic evaluation. Before image collection, dogs were introduced in a room with a controlled temperature, set at 21 °C. They were allowed to walk calmly to adjust to room temperature for 30 min. During the actual image collection, animals were positioned standing in an upright position, as symmetrically as possible. When required, the dog’s trainer assisted in maintaining the animal’s position. They were allowed to touch the dog’s abdomen but not its torso. Each dorsoventral image included the last lumbar vertebra area to the first coccygeal vertebra (Fig. 1). This procedure has high repeatability between observers and cameras [13]. For the lateral views, the greater trochanter was located and placed in the image’s center (Fig. 2). Figure 3 shows a dog at the time of initial evaluation and 30 days post-treatment. No fur clipping was performed before image collection since it can be harmful for thermographic evaluation, affecting reading stability for at least 60 min after clipping [14]. Image settings were adjusted to include a range of 15–40 °C and emissivity of 0.98. Thermographic images were analyzed with Tools (FLIR Systems, Inc), a Rainbow HC color pallet was selected, and equal-sized temperature boxes were placed on the hip joint’s anatomical area. Mean and maximal temperatures were determined.

After image collection, handlers received the published instructions for CBPI and COI and completed an online copy of each for them. These were completed in sequence by the same handler, which was blinded to the group his/her dog was assigned, in each of the follow-up assessments, without knowledge of their previous answer. Weight-bearing distribution was obtained with the Companion Stance Analyzer (LiteCure LLC, Newark, Delaware, United States). The equipment was placed on a flat surface in the center of a room, at least 1 m from the walls, calibrated at the beginning of each day, and zeroed before each evaluation moment. Animals were encouraged by their handlers to stand on to the weight distribution platform, with one foot on each quadrant. Gentle restraint was used to maintain the patient’s head in a natural, forward-facing position. For each patient, 20 measurements were conducted, and a mean value was obtained. Normal weight distribution for each limb is considered 20% of the total weight [41], and we also considered the deviation from this value, obtained by subtracting the weight-bearing of the limb to 20. Additionally, a left-right symmetry index (SI) was calculated, using the following formula: SI = [(WB_R_-WB_L_)/((WB_R_ + WB_L_)× 0.5)]× 100 (WB_R_ = weight-bearing of the right pelvic limb and WB_L_ = weight-bearing for the left pelvic limb) [32, 65]. Negative symmetry index values were transformed to positive values. Radiographic images were conducted under light sedation, using a combination of medetomidine (0.01 mg/kg) and buthorphanol (0.1 mg/kg), given intravenously. A ventrodorsal extended view was obtained, as described elsewhere [43]. Since sedation can influence blood circulation and body temperature, this procedure was conducted after weight-bearing and digital thermography evaluations. On day 0, the body condition score was determined, according to the Laflamme scale [42].

Normality was assessed with a Shapiro-Wilk test. Mean and maximal values obtained on the dorsoventral view at the initial evaluation and during the follow-up period were compared with those obtained on the lateral view with ANOVA, followed by an LSD post-hoc test, or the Wilcoxon test, as appropriate for the data distribution. Correlations were assessed with the Spearman correlation coefficient. Results were analyzed with IBM SPSS Statistics version 20, *p* < 0.05.

## Data Availability

The datasets used and/or analysed during the current study are available from the corresponding author on reasonable request.

## Abbreviations

CBPI: Canine Brief Pain Inventory
COI: Canine Orthopedic Index
DV: Dorsoventral view
OA: Osteoarthritis
PIS: Pain Interference Score
PSS: Pain Severity Score
